# Physicians' attitudes about obesity and their associations with competency and specialty: A cross-sectional study

**DOI:** 10.1186/1472-6963-9-106

**Published:** 2009-06-24

**Authors:** Melanie Jay, Adina Kalet, Tavinder Ark, Michelle McMacken, Mary Jo Messito, Regina Richter, Sheira Schlair, Scott Sherman, Sondra Zabar, Colleen Gillespie

**Affiliations:** 1 Division of General Internal Medicine, New York University School of Medicine, New York, NY USA; 2 Department of Pediatrics, New York University School of Medicine, New York, NY USA; 3 VA New York Harbor Healthcare System, New York, NY USA

## Abstract

**Background:**

Physicians frequently report negative attitudes about obesity which is thought to affect patient care. However, little is known about how attitudes toward treating obese patients are formed. We conducted a cross-sectional survey of physicians in order to better characterize their attitudes and explore the relationships among attitudes, perceived competency in obesity care, including report of weight loss in patients, and other key physician, training, and practice characteristics.

**Methods:**

We surveyed all 399 physicians from internal medicine, pediatrics, and psychiatry specialties at one institution regarding obesity care attitudes, competency, including physician report of percent of their patients who lose weight. We performed a factor analysis on the attitude items and used hierarchical regression analysis to explore the degree to which competency, reported weight loss, physician, training and practice characteristics explained the variance in each attitude factor.

**Results:**

The overall response rate was 63%. More than 40% of physicians had a negative reaction towards obese patients, 56% felt qualified to treat obesity, and 46% felt successful in this realm. The factor analysis revealed 4 factors–*Physician Discomfort/Bias, Physician Success/Self Efficacy, Positive Outcome Expectancy*, and *Negative Outcome Expectancy*. Competency and reported percent of patients who lose weight were most strongly associated with the *Physician Success/Self Efficacy *attitude factor. Greater skill in patient assessment was associated with less *Physician Discomfort/Bias*. Training characteristics were associated with outcome expectancies with newer physicians reporting more positive treatment expectancies. Pediatric faculty was more positive and psychiatry faculty less negative in their treatment expectancies than internal medicine faculty.

**Conclusion:**

Physician attitudes towards obesity are associated with competency, specialty, and years since postgraduate training. Further study is necessary to determine the direction of influence and to explore the impact of these attitudes on patient care.

## Background

Despite the high prevalence of obesity[1] and its associated harmful health effects [2] physicians frequently fail to counsel patients about nutrition and weight management [3-7] and frequently report and demonstrate a lack of training and competence in obesity management [8-13]. Clinicians who learn good obesity screening and counseling practices in residency are more likely to report that they always discuss diet or exercise with obese patients [12]. Therefore training of physicians has the potential to impact patient outcomes.

Attitudes, or "individual's positive or negative evaluation of performing a behavior [14]" may also contribute to insufficient or ineffective counseling practices. Major theories predicting practice behavior postulate that attitudes are important influences on the decision to engage in a defined behavior [14,15]. Attitudes can serve to motivate a provider to take action and encourage a physician to feel capable of tackling a particular behavior (e.g., self-efficacy, perceived behavior control [16]). Indeed, higher self efficacy in delivering preventive care services was predictive of preventive counseling in pediatricians [17]. Attitudes also may instill in a physician the belief that the behavior in question is worth doing because it will produce the intended outcomes (e.g., perceived benefits, outcome expectancies [14]).

Thus, physicians' attitudes about obesity may affect their practice. Studies have shown that physicians' attitudes toward obese patients can be negative, e.g., some primary care physicians rate obese patients negatively with respect to their likelihood of following advice, believe that obese patients are less likely to benefit from counseling, and report less desire to help obese patients [18], and therefore may decrease physicians' motivation to counsel such patients. Even health professionals specializing in obesity have implicit anti-fat bias and are more likely to automatically associate 'fat people' with negative stereotypes than 'thin people' [19,20]. Physicians' attitudes and beliefs about their own general efficacy in treating obesity may also influence their treatment of patients. In one study, physicians rated obesity treatment as less effective than therapies for 9 out of 10 chronic conditions, and only 14% agreed that they were usually successful in helping obese patients lose weight [21]. In another study, 31% of internal medicine residents believed that treating obesity is futile and only 44% felt qualified to treat obese patients [11]. We have found no studies of direct impact of negative physician attitudes on obesity care.

While attitudes are thought to influence physician behavior with regards to obesity counseling, we know very little about how these attitudes are formed or influenced. Social cognitive theory and the theory of planned behavior suggest that competence (skills or prior performance) is an important influence on attitudes [14,15]. A few studies have tested this hypothesis in obesity with mixed results: one study showed that perceived competency was correlated with comfort level in treating pediatric obesity and the belief that obesity is a treatable condition [22], while another study did not find any link between knowledge (an essential determinant of competence) and attitudes [11].

Models of behavior change also suggest that social and environmental factors play an important part in shaping attitudes. For physicians these include individual physician characteristics (e.g., age, gender) [20,23,24], training (e.g., specialty, when trained, exposure to role models)[25]. Practice contexts (e.g., type of patients seen, public vs. private setting, inpatient vs. outpatient) may also influence attitudes. With the exception of physician bias [19,26], few studies have characterized physicians' attitudes about obesity with regards to gender, specialty, training and practice characteristics. Further, while several studies have described individual physicians' attitudes about obesity [22,27-29], none have characterized how these attitudes relate to each other.

Additional studies are needed to elucidate the relative contribution of individual competence, physician, training, and practice variables on attitude formation. This is necessary in order to design more carefully targeted interventions and ultimately to be able to assess the impact of these attitudes on actual physician practice and patient outcomes.

As part of a needs assessment for a faculty development project to improve obesity care, we surveyed physician faculty members about their attitudes, obesity counseling competency (including report of percentage of their obese patients who lose weight), training, and practice characteristics. The first aim of this study was to understand and classify the nature of physician attitudes regarding obesity and examine how they differ by specialty. The second aim of this study was to better understand how competency in obesity counseling, physician characteristics, training, and practice characteristics are related to attitudes. The final aim was to explore the relative association of each of these on physician attitudes.

## Methods

### Participants and Study Implementation

We sent a confidential online survey via email to 399 New York University School of Medicine faculty members identified from departmental lists. Responses were automatically loaded into a database. Up to 8 reminder emails, over a 4-month period, were sent to non-respondents to encourage them to complete the survey. This sample represented all Internal Medicine (IM), Pediatric (Peds), and Psychiatric (Psych) faculty excluding 77 who were no longer at the institution, 11 collaborators in this study, 38 without working emails, and 24 non-physicians or neonatologists. These three groups were chosen because faculty leaders from each were participating in a collaborative project to improve obesity training across three specialties–all of which are centrally involved in medical and behavioral management of obese patients.

### Survey Measures

Attitudes toward specific aspects of obesity counseling were derived from the literature [11,21,22] and fell within 3 broad target areas: Attitudes toward obese people (e.g., "I feel uncomfortable when examining an obese patient" and "It is difficult for me to feel empathy for an obese patient" [21]); attitudes toward treatment (e.g., "Obesity is a treatable condition" [22]; "Most obese patients will not lose a significant amount of weight" [21]); and attitudes toward obesity as a disease (e.g., "Obesity is primarily caused by behavioral factors"[11]). We generated 37 items through this process, reducing the total to 24 by eliminating overlapping or redundant items. We then invited local faculty to help us choose the final 12 items by expert consensus. These items were arranged in random order and agreement with them was assessed on a 4-point Likert scale (Disagree Strongly [1]; Disagree Somewhat [2]; Agree Somewhat [3]; and Agree Strongly [4]). These attitude items were then piloted with 67 residents, and we found that no changes were needed.

We generated items assessing physicians' attitudes towards obese patients and their perceived competency to perform 15 different obesity counseling skills. Competence was broken down into 5 specific aspects of obesity counseling and treatment based on the 5A's of behavioral counseling recommended by the United States Preventative Services Task Force [30]. The 5As framework guides the physician to **A**ssess risk, current behavior, and readiness to change, **A**dvise change of specific behaviors, **A**gree and collaboratively set goals, **A**ssist in addressing barriers and securing support, and **A**rrange for follow-up [30,31]. This scale has been described in detail elsewhere [9]. In addition, we included an assessment of physicians' perception of the percent of their obese patients who lose weight as an indirect proxy of patient outcome.

The survey also assessed physicians' gender, training variables (specialty, years since residency), and practice characteristics (time spent in outpatient vs. inpatient, and public vs. private settings, percent of patients in practice that are obese) to capture possible social and environmental influences on attitudes. Degree of involvement in a public setting care was hypothesized to involve more complex and challenging patient populations and degree of outpatient practice and percent of obese patients were thought to influence physicians' experience in and opportunities for providing obesity counseling.

### Analyses

We used factor analysis (principal components) to identify underlying structure among the 12 attitudes items and to reduce data for subsequent analyses. Factor structures were rotated (varimax and therefore orthogonal) for ease of interpretation. Factors were extracted based on minimum item loadings of .40, examination of scree plots, and simplicity of factor structure (loading on only one factor). We generated factor scores for each respondent by averaging across attitude items for each factor. Items with negative factor loadings were reverse-coded.

We averaged competency in obesity counseling and treatment across all 15 items to arrive at a single overall competency score and created 5 sub-scores by averaging items within each of the 5A's competency areas [9]. Our patient outcome was physician-reported percent of patients in one's practice who lose weight. We compared competency scores and percent of patients who lose weight (multiple dependent variables) across specialties (one independent variable) using separate multivariate ANOVAs with post hoc Tukey tests to assess the significance of differences between specialties.

Differences in attitudes, competency, and the other characteristics were explored using Chi Square analyses for categorical variables and one-way ANOVAs for continuous variables. Tukey's post hoc tests were used to identify significant pair-wise comparisons when overall ANOVA F values were significant (p < .05). Bivariate correlations (Pearson's *r*) were calculated to determine associations between attitude factors and physician characteristics, training, practice characteristics, and competencies. We then used hierarchical regression to explore associations between each of the presumed independent variables (competency, training, physician characteristics, and practice characteristics) and each of the attitude factors (treated as a dependent variable despite the cross-sectional nature of our study) with all of the variables in the model. These analyses sought to assess the contribution of each core variable to explaining variance in the specific attitude while controlling for all other variables and were run separately for each of the four attitudes. This study was approved by the Institutional Review Board at New York University School of Medicine.

## Results

### Participants

We received responses from 250 physicians (63%), which are summarized in Table 1. Response rates did not differ by specialty and respondents did not differ from non-respondents with regards to gender (the only socio-demographic variable gathered). A greater percentage of pediatricians were women, although the difference was not significant. Physicians spent about 25–50% of their time providing care in public settings and an average of about 20 hours per week seeing outpatients. Internal medicine faculty reported having the highest rate of obesity in their patient population. Overall, physicians felt fairly competent in providing obesity counseling and reported that an average of 14% of their obese patients lost weight. Psychiatry faculty felt less competent in Assess, Advise, and Agree counseling skills than either Internal Medicine or Pediatrics faculty. Not surprisingly, pediatric faculty reported the lowest percentage of patients who lost weight in their practice.

**Table 1:** Characteristics of the sample (n = 250)

**Variables**	**Specialty**				
	**Full Sample**	**Internal Medicine**	**Pediatrics**	**Psychiatry**	***Significance***^1^

% (n)	100% (250)	37% (93)	24% (59)	39% (98)	

**Physician Characteristics**					

% (n) Female	48% (121)	39% (36)	56% (33)	44% (43)	X^2 ^= 4.39 p = .112

**Training Characteristics**					

Mean (SD) Yrs Since Residency	14.4 (11.4)	13.7 (11.5)	13.9 (10.7)	16.8 (12.3)	F = 1.39 p = .252

**Practice Characteristics**					

Time Spent Providing Direct Care in Public Outpatient Setting^2^	2.5 (1.2)	2.7 (1.4)	2.6 (1.3)	2.3 (1.5)	F = 1.35 P = .26

Mean (SD) Hrs/Wk See Outpatients	22.0 (14.8)	21.5 (14.8)	24.1 (17.3)	21.4 (13.5)	F = .58 p = .562

Mean (SD) % of Pts in Practice Obese	21.9 (16.5)	28.4 (15.8)	18.2 (16.3)	18.0 (15.4)	F = 11.22 P < .001 Peds, Psych<IM

**Perceived Obesity Counseling Skills**^3^					

Mean (SD) Assess	2.9 (.6)	3.0 (.5)	3.1 (.6)	2.8 (.7)	F = 6.66 p = .002 Psych<IM, Peds

Mean (SD) Advise	2.9 (.7)	3.1 (.6)	3.1 (.6)	2.8 (.7)	F = 5.95 p = .003 Psych<IM, Peds

Mean (SD) Agree	2.8 (.8)	2.9 (.7)	2.8 (.8)	2.6 (.8)	F = 5.32 p = .006 Psych<IM, Peds

Mean (SD) Assist	2.6 (.8)	2.7 (.7)	2.6 (.8)	2.6 (.8)	F = .16 p = .85

Mean (SD) Arrange	2.9 (.7)	2.8 (.7)	3.0 (.7)	3.9 (.7)	F = 2.63 p = .074

Mean (SD) Reported % of Pts in Practice Lose Weight	14.4 (11.4)	15.2 (15.7)	7.5 (9.0)	16.5 (18.3)	F = 4.44 p = .013 Psych, IM>Peds

^1^Chi square analyses conducted for categorical variables, one way ANOVAs conducted for continuous variables. Tukey's post hoc tests used to identify group differences when overall F test was <.05.

^2 ^5-point scale: 1 = No time spent, 2 = up to 25% of effort, 3 = 25% – 50% effort, 4 = 50% – 75%, 5>75% effort

^3 ^Average of questions corresponding to each 5A's category using 4-point scale: 1 = Know very little about and not able to perform; 2 = Know something about and somewhat able to perform; 3 = Able to perform well; and 4 = Able to teach others how to perform

### Attitude items and factor analysis

Table 2 shows the responses to the individual attitude questions. Of note, 45% of physicians agree that they have a negative reaction to the appearance of obese individuals which did not differ between specialties. Only slightly more than half felt qualified to treat obese patients with psychiatry faculty feeling significantly less qualified than the other specialties (p = .002). More than half did not feel successful at treating obese patients, with no differences seen between specialties. The majority of physicians felt that treating obese patients is very frustrating with Psychiatry faculty being least likely to agree (p < .001).

**Table 2:** Factor analysis of attitudes and physician agreement with attitude items (n = 250)

			**Loadings**				**Distribution of Responses**	
**Factor Name**	**% Var**	**Attitude Item**	**1**	**2**	**3**	**4**	**% Agree**	**% Disagree**
Factor 1 Physician Discomfort/Bias	25%	I feel uncomfortable when examining an obese patient	**.80**	-.25	-.02	-.01	18	82
		It is difficult for me to feel empathy for an obese patient	**.77**	-.16	.06	.10	8	92
		I have negative reactions towards the appearance of obese patients	**.76**	-.06	-.01	.12	45	55

Factor 2 Physician Success/Self Efficacy	16%	I feel qualified to treat obese patients	-.11	**.84**	.09	.21	55	45
		I have been successful in treating patients for obesity	-.14	**.72**	.05	-.17	46	54
		The best role for a physician in weight management is to provide treatment rather than referral	-.18	**.56**	-.06	-.22	88	12

Factor 3 Positive Outcome	11%	Most obese patients could reach a normal weight (for height) if motivated	.06	.02	**.77**	-.08	38	61
Expectancy		Obesity is primarily caused by behavioral factors	-.04	-.19	**.72**	.30	33	67
		Obesity is a treatable condition	.01	.22	**.70**	-.20	92	8

Factor 4 Negative Outcome	11%	Most obese patients will not lose a significant amount of weight	-.04	-.23	-.18	**.79**	49	51
Expectancy		Treating obese patients is very frustrating	.38	.09	.12	**.70**	66	34

Using a factor loading of .40 or greater as the cut-off (all factor loadings exceeded .56), we identified 4 factors accounting for more than 60% of the variance as shown in Table 2. One question ("Most obese patients are well aware of the health risks of obesity") factored separately and was excluded from further analysis. Another item ("The best role for a physician in weight management is to provide referral rather than treatment") was reverse coded since it negatively loaded onto the factor.

Table 3 shows differences in mean attitude factor scores by specialty and gender. Attitudes related to outcome expectancies differed by specialty with pediatric faculty reporting more positive outcome expectancies (mean = 2.85 on a 4-point scale, SD = .53) than internal medicine (mean = 2.49, SD = .49) or psychiatry (mean = 2.53, SD = .52) (p < .001) and psychiatry faculty reporting less agreement with negative outcome expectancies (mean = 2.38, SD = .55) than internal medicine (mean = 2.75, SD = .63) or pediatric (mean = 2.90, SD = .61) faculty (*p *< .001). Women had more positive outcome expectancies than men (mean = 2.68, SD = .52 vs. mean = 2.49, SD = .54, p = .007).

**Table 3:** Differences in Mean Attitude Factor Scores *by Background Characteristics

**Background Characteristics**	Physician Discomfort/Bias		Physician Success		Positive Outcome Expectancies		Negative Outcome Expectancies	
Specialty								

Internal Medicine	1.77 (.58)	P = .085	2.74 (.58)	P = .512	2.49 (.49)	P < .001	2.75 (.63)	P < .001
							
Pediatrics	1.64 (.52)		2.73 (.60)		2.85 (.53)	Peds > IM, Psych	2.90 (.61)	Psych < IM, Peds
							
Psychiatry	1.87 (.65)		2.65 (.56)		2.53 (.52)		2.38 (.55)	

Gender								

Male	1.78 (.58)	P = .869	2.70 (.56)	P = .894	2.49 (.53)	P = .007	2.68 (.62)	P = .197
							
Female	1.76 (.62)		2.71 (.57)		2.68 (.52)		2.58 (.65)	

*4-point Likert Scale (Disagree Strongly [1]; Disagree Somewhat [2]; Agree Somewhat [3]; and Agree Strongly [4])

Table 4 shows correlations between the attitude factors and physician competence in the 5A's categories, training (years since residency), and practice characteristics. Years since residency was negatively correlated with positive outcome expectancy and positively correlated with negative outcome expectancy. Amount of time spent in outpatient practice was positively correlated with physician success although time spent in public care setting was negatively associated with physician success. Only two attitude factors, *Physician Discomfort/Bias *and *Physician Success/Self Efficacy *were correlated with perceived 5A's competencies. Only one attitude, *Physician Success/Self Efficacy*, was associated with report of % of obese patients who lose weight. *Physician Discomfort/Bias *was negatively correlated with Assess, Advise, Agree, and Arrange competencies. *Physician Success *was positively (and significantly) correlated with all 5 counseling competencies as well as % of obese patients who lose weight.

**Table 4:** Correlations (Pearson's r) between attitudes and training, practice Characteristics, and obesity counseling competencies (n = 250)

	**Physician Discomfort/Bias**	**Physician Success**	**Positive Outcome Expectancies**	**Negative Outcome Expectancies**
**Training Characteristics**				

Years Since Residency	-.08	.10	-.18**	.15*

**Practice Characteristics**				

Time Spent Providing Direct Care in Public Outpatient Setting^1^	.04	-.18**	.09	.06

Hours/Wk See Outpatients	-.06	.16*	.04	.01

% of Patients in Practice Obese	-.01	-.02	-.02	.12

**Perceived Obesity Counseling Skills**				

Mean (SD) Assess Score	-.25***	.55***	.00	-.01

Mean (SD) Advise	-.18**	.47**	-.01	.09

Mean (SD) Agree	-.21**	.57***	-.02	-.08

Mean (SD) Assist	-.13^	.37***	.08	-.11

Mean (SD) Arrange	-.14*	.42***	.04	-.06

% of Obese Patients Lose Weight	-.14^	.30***	-.09	-.14^

^1 ^5-point scale: 1 = No time spent, 2 = up to 25% of effort, 3 = 25% – 50% effort, 4 = 50% – 75%, 5>75% effort

*P < .05, **p < .01, ***p < .001

^P = .054–.056

### Regression analyses

Table 5 shows four regression models with each of the attitude factors as dependent variables. Standardized betas are reported to show magnitude and direction of the association with all other independent variables in the model. Our model explained a moderate amount of variance (34%, p < .001) in Physician Success/Self Efficacy. Significant variables included the 'Assess' and 'Agree' competency scores and reported % of obese patients who lose weight.

**Table 5:** Four separate regression analyses predicting each attitude factor score (n = 250)

**Dependent Variables**	***Attitudes***							
	
	**Physician Discomfort/Bias**		**Physician Success**		**Positive Outcome Expectancies**		**Negative Outcome Expectancies**	
***Independent Variables***	***Std Beta***	***p***	***Std Beta***	***p***	***Std Beta***	***p***	***Std Beta***	***p***

**Perceived Obesity Counseling Skills**								

Assess	**-.21**	**.045**	**.29**	**.001**	**-.20**	**.048**	-.06	.555

Advise	.06	.556	-.04	.655	.06	.569	**.24**	**.012**

Agree	-.10	.309	**.31**	**.000**	-.04	.676	**-.23**	**.016**

Assist	.04	.654	.01	.922	**.18**	**.023**	-.09	.227

Arrange	.04	.692	.03	.670	.06	.465	.06	.435

% of Obese Pts Lose Weight	-.11	.102	**.11**	**.047**	.00	.958	-.10	.106

**Physician Characteristics**								

Female	-.03	.719	.00	.969	.08	.250	-.05	.441

**Training Characteristics**								

Specialty: Pediatrics	-.10	.190	.00	.969	**.27**	**.000**	.08	.281

Specialty: Psychiatry	.02	.793	.03	.994	-.01	.863	**-.27**	**.000**

Yrs Since Residency	-.07	.340	.04	.664	**-.22**	**.001**	**.13**	**.049**

**Practice Characteristics**								

Time Spent in Direct Pt Care in Public Outpatient Setting^1^	.00	.998	-.06	.315	.05	.734	.01	.854

Hours/Wk See Outpatients	-.02	.757	.10	.089	.04	.563	-.02	.775

% of Pt in Practice Obese	-.00	.963	-.00	.951	-.03	.646	.11	.122

**Total Variance (R^2^) Explained**	**R^2 ^= 3.7% p = .046**		**R^2 ^= 33.6% p < .001**		**R^2 ^= 9.8% p < .001**		**R^2 ^= 15.6% p < .001**	

^1 ^5-point scale: 1 = No time spent, 2 = up to 25% of effort, 3 = 25% – 50% effort, 4 = 50% – 75%, 5>75% effort

The only variable significantly associated with *Physician Discomfort/Bias*, when all variables were in the model, was the "Assess" competency score. Greater ability in assessment was associated with less discomfort/bias. However, the entire model only explained 4% of the variance

Pediatrics faculty held stronger positive outcome expectancy beliefs and psychiatry faculty held less strong negative outcome expectancies than internal medicine faculty. Recently trained physicians held more positive and less negative outcome expectancy beliefs than those physicians whose residency training was further back in time. With all variables in the model, competence was associated with outcome expectancies (bivariate relationships were not significant) such that competence in Assisting in addressing barriers and securing support was positively and competence in Assessing negatively associated with positive treatment expectancies. Similarly, competence in one of the 5 A's (Advise) was positively associated with negative outcome expectancies while another (Agree) was negatively associated. This pattern of positive and negative associations probably explains why significant bivariate relationships were not found.

## Discussion

This is the first study that we know of to explore physicians' attitudes towards obesity with regards to competency, perceived patient outcome, physician, and practice characteristics. Based on the Theory of Planned Behavior and Social Cognitive Theory, we anticipated that perceived competency and skills would relate to physicians' attitudes. Physician Success/Self-efficacy was the attitude factor most associated with competency and reported patient weight loss. Whether this association is causal needs to be determined by prospective studies looking at actual physician performance and true patient outcomes. The direction of the association is unclear in our analysis. We cannot be certain whether physician self efficacy leads to improvements in competency and reported patient outcomes or vice-versa. Further, these associations may be artificial if, for instance, the *Physician Success/Self Efficacy *attitude factor and self-reported competency items are both measuring the same underlying variables. Indeed, the definition of self efficacy is "the belief that one can successfully execute the behavior required to produce the outcome [15]" which is similar to perceived competency. However, we believe that our competency and self-efficacy scales are, in fact, distinct. The competency items we used measured physicians' self-reported ability to perform specific 5A's related tasks while the *Physician Success/Self Efficacy *attitude factor measured a more global belief in their qualifications and success in treating obesity.

We found significant associations between the specific 5A's competency categories and particular attitude factors. The "Assess" competency score was negatively associated with *Physician Discomfort/Bias *and positively associated with *Physician Success/Self Efficacy *suggesting that physicians with lower competency assessing risk in obese patients may have more overall discomfort treating obese patients and lower self efficacy. The "Agree" competency score was positively associated with physician self efficacy indicating that physicians who are proficient in mutual goal-setting with patients may possess greater self-efficacy. Finally, the "Assist" competency score was positively associated with *Positive Outcome Expectancy *indicating that the ability to address barriers to weight loss and motivational interviewing may contribute to higher expectations that obese patients will be successful at losing weight. Overall, this pattern of results suggests that particular skills may be associated with the development and maintenance of particular attitudes. Since the 5A's competencies represent teachable skills, training may improve physician attitudes ultimately leading to better care for obese individuals.

Attitudes and competency varied by specialty. Psychiatrists reported being less competent performing many of the 5A's-related skills than the other specialties. This may be due to a lack of experience screening and counseling for obesity. Indeed, the psychiatrists indicated that only 19% of their patients were obese even though the prevalence of obesity in the psychiatric population is known to be higher than the general population [32,33] which indicates that they may not be sufficiently recognizing obesity in their patients. However, psychiatrists also have less negative outcome expectancy than the other specialties which could facilitate receptiveness to acquire obesity counseling skills. We believe that psychiatrists should be trained to counsel patients to lose weight since many psychotropic medications cause weight gain, stimulate appetite, and increase cardiovascular risk [34,35]. Further, obesity can be thought of as a neurobehavioral disorder [36] that psychiatrists' training should enable them to treat. Being a pediatrician was associated with having a higher positive outcome expectancy despite the fact that pediatricians reported a lower percentage of patients losing weight in their practice. Further examination of the relationship between specialty and attitudes is needed.

We also explored how physicians' gender is associated with obesity attitudes. Females were more likely to have higher positive outcome expectancies than males in bivariate analysis. However, when we controlled for other physician and practice characteristics, gender was no longer associated with any of the attitude factors. Teachman et al. also did not find gender differences in either implicit or explicit bias in physicians [24]. However, Schwartz et al. showed that being a male physician was associated with lower levels of bias [26]. Future studies should further explore the potential impact of gender on physician attitudes.

In addition to gender, outcome expectancies seem to be associated with the number of years elapsed since residency/postgraduate training. Physicians with greater time lapse since training (and thus presumably more experience) had higher *Negative Outcome Expectancy *and lower *Positive Outcome Expectancy*. We have at least two possible explanations for this. First, physicians may become disillusioned with years of attempted treatment of obese patients in the face of modest weight loss or even weight gain. Second, obesity management training may be different for recent graduates who practice in an era in which it is widely accepted that even modest weight loss should be considered a successful outcome [37]. Thus, they may view success differently than their more senior peers, which may be reflected in more positive outcome expectancies. However, both of these hypotheses are speculative and these results need to be replicated. Indeed, Ferrante et al. found that older physicians were actually *less *likely to agree that treatment of obesity is ineffective than younger physicians [38].

Unfortunately, our models only account for 4–33% of the variance in physicians' attitudes about obesity suggesting that other unmeasured factors are more important influences on physician attitudes. For example, physicians' health behaviors potentially play a significant role in shaping their attitudes about obesity. Several studies show that personal health promotion behavior in physicians is a consistent and powerful predictor of attitudes and/or professional practice towards obesity-related behavior change counseling [39-43]. Physicians who try to exercise more and maintain a healthy diet are significantly more likely to discuss exercise and weight with their patients and report greater counseling self-efficacy [44]. Thus, a limitation of our study is that we did not include physicians' own behaviors in the survey.

There are several other limitations as well. We relied on physician self-report for competency and patient weight loss items rather than direct measurement. Several studies have suggested that physicians may not be reliable evaluators of their competency [45]. However, our measure may be more sensitive than other measures of competency as it evaluates 15 behaviorally-specific skills. We are in the process of evaluating this measure against actual physician performance. Further, it is unclear how accurately physicians were able to estimate how many of their obese patients lose weight. All the physicians were from the faculty at New York University School of Medicine which may limit generalizability. However, the physicians surveyed practice in a wide array of settings–in both the public and private sector, hospital and clinic settings–and represent 3 different medical specialties. Our findings are also limited in that attitudes items measured do not represent the full spectrum of attitude measures in the literature.

Our study suggests avenues for future research. This work will help us to design future studies to assess the impact of physician attitudes on performance and patient care. Observed structured clinical exams using standardized patients (OSCEs) are often used to measure physician performance, and we plan to measure how attitudes relate to OSCE performance. Further, we propose doing chart reviews and patient exit interviews to help determine how physician attitudes relate to real rather than perceived outcomes in overweight and obese patients such as eating and exercise behaviors and weight loss.

## Conclusion

There is strong evidence that physicians do not counsel obese patients adequately and attitudes may be one reason for this deficiency. This study suggests specialty, years since residency, competency, and reported patient weight loss may influence how physicians' attitudes are formed. These factors all may ultimately affect patient care. Competency and reported patient weight loss were most strongly associated with *Physician Success/Self efficacy*. This study provides a framework to evaluate other predictors of physicians' obesity attitudes as well as the contribution of these attitudes to patient care and outcomes.

## Competing interests

The authors declare that they have no competing interests.

## Authors' contributions

MJ participated in drafting the manuscript, participated in the design of the measures and design of the study. CG and AK participated in drafting the manuscript, participated in the design of the measures and design of the study. TA participated in the design of the measures and drafting the manuscript. SS, MM, SZ, and RR participated in the design of the study. All the authors read and approved the final manuscript.

## Authors' Information

Melanie Jay, MD, MS is a general internist and Clinical Assistant Professor at New York University School of Medicine. She is a member of Research on Medical Education Outcomes Team (ROMEO). Colleen Gillespie, PhD is a community psychologist and Assistant Professor of Medicine. She is a member of ROMEO. Sheira Schlair, MD, MSc is a general internist and clinical instructor at New York University School of Medicine. She is a member of ROMEO. Tavindar Ark has a Masters degree in psychology and is a Research Associate at New York University School of Medicine. She is a member of ROMEO. Scott Sherman MD, MPH is a general internist and geriatrician. He is an Associate Professor of Medicine at New York University School of Medicine. Michelle McMacken MD, MPH is a general internist and a Clinical Assistant Professor at New York University School of Medicine. She is director of the Weight Management Program at Bellevue Hospital. Mary Jo Messito, MD is a pediatrician and an Assistant Clinical Professor at New York University School of Medicine. Sondra Zabar, MD is an associate professor of medicine at New York University School of Medicine and the program director of the NYU primary care residency program. She is a member of ROMEO. Adina Kalet, MD, MPH is a general internist and Professor of Medicine at New York University School of Medicine. She has done extensive research in medical education and is the leader of ROMEO.

## Pre-publication history

The pre-publication history for this paper can be accessed here:


